# Big wigs and small wigs: Time, sex, size and shelter affect cohabitation in the maritime earwig (*Anisolabis maritima*)

**DOI:** 10.1371/journal.pone.0185754

**Published:** 2017-10-02

**Authors:** Nicole L. Hack, Vikram K. Iyengar

**Affiliations:** 1 Department of Biological Sciences, California Polytechnic State University San Luis Obispo, San Luis Obispo, California, United States of America; 2 Department of Biology, Villanova University, Villanova, Pennsylvania, United States of America; Fundacao Oswaldo Cruz, BRAZIL

## Abstract

Animal aggregations can occur for a variety of abiotic factors, such as resource limitation, or biotic factors, including group foraging and protection from predators. In our study, we examined whether time, sex, body size or shelter availability affected aggregation behavior of the maritime earwig, *Anisolabis maritima* (Order Dermaptera), an insect found globally at high densities under driftwood. Specifically, we monitored the distribution of two individuals in arenas with either two shelters (no habitat limitation) or one shelter (habitat limitation) to determine their propensity for cohabitation at times of peak activity and times of quiescence. Females, whose high levels of aggression are often associated with maternal care, were particularly averse to cohabitation, whereas males were generally more tolerant of other earwigs. Females initially preferred not to cohabitate when placed with a male, but were more tolerant of cohabitation later, regardless of the number of shelters. Same-sex pairs, on the other hand, were less likely to cohabitate with only one shelter present, but males were again more tolerant of conspecifics than females regardless of habitat limitation. When competition for one shelter did not lead to cohabitation, the lone occupant was more likely to be the larger individual in same-sex trials and females in mixed-sex trials. Understanding the tolerance for close proximity under these varying conditions may provide insight into aggregative behavior and spatial distribution patterns in the maritime earwig.

## Introduction

Animals are often found in aggregations due to an individuals’ choice to cohabitate or a tolerance of others [1,2]. Specifically, social factors that may select for aggregative behavior include protection in numbers [3,4], and other potentially kinship-related benefits [5,6]. Groups can also form in correspondence with mate choice, where highly sought-after females obtain groups of courters [7], alpha males form harems of females [8], or males showcase their prowess to females in leks [9]. On the other hand, resources may select for living in close proximity, as aggregations can also be due to exploitation of common resources and may not indicate gregariousness [10]. Regardless, living in close proximity to conspecifics is relatively rare given that the benefits must outweigh the costs of increased competition for food and mates [4]. Given the numerous potential causal factors at play, we manipulated individual and environmental parameters to understand the aggregative behavior of an insect that is found at high densities.

*Anisolabis maritima*, the maritime or seaside earwig (Family Anisolabididae), is an insect with a worldwide distribution along both temperate and tropical coastal habitats [11,12]. While other members of the order Dermaptera possess hardened posterior forceps used in prey capture of small arthropods [13], *A*. *maritima* is characterized by a unique directional asymmetry in the male’s forceps, which may in part explain sex differences in how forceps are used in aggressive encounters with conspecifics [14,15]. Males tend to engage in non-lethal pinching of a rival’s abdomen (perhaps as a ritualistic means to assess strength; [15]), whereas females use their forceps to jab at and cut conspecifics into pieces [16]. Additionally, female earwigs are larger than males, presumably due to a fecundity advantage seen in many arthropods [17] and the fact that females engage in maternal care, aggressively guarding eggs and juveniles for up to 28 days [16,18,19].

Although this beach-dwelling insect is abundant and large in size (15 to 32 mm), *A*. *maritima* are inconspicuous because they spend most of their time in aggregations under driftwood [13,20]. The aggregations on beaches in the San Juan Islands, Washington, appear mostly restricted to an area about 3 meters above the high-tide line, where they can avoid getting water-logged by the tide but also avoid niche overlap with ants that prefer drier areas (pers. obs.). Among the populations we sampled, maritime earwig numbers can vary widely, but we have observed natural densities as high as 2.75 earwigs/100 cm^2^ with a nearest average nest distance of 5.85 +/- 0.61 cm. In populations in California, Miller *et al*. found about 39% of nests were within 20 cm of another conspecific and 9% were within 0.5 cm [16]. Across their range, these earwigs are typically found in close proximity to each other beneath driftwood, a critical, limited resource that allows them to reduce the risks of predation and desiccation and emerge only at night to forage [13,20].

These aggregations were previously proposed to be present due to habitat limitations [16], yet it can be hard to decipher between gregariousness and the pressure to share common resources. In this study, we measured the likelihood of cohabitation of different sized pairs of maritime earwigs with either one or two shelters to determine the roles that sex, relative body size, and shelter limitation play in their aggregation behavior. We also measured the cohabitation patterns both at night, their peak activity time, and the next day, their quiescent period, to determine if there was a temporal change in their tolerance of each other [21]. Overall, we hypothesized that females, who are typically more aggressive due to maternal care, would be less likely to cohabitate compared to males, who typically engage in non-lethal fighting. We also expected relative size to play a role in competition because larger individuals tend to dominate in competition for food [15]. Given that earwigs are only found in the daytime under driftwood, we posited that the importance of cover would result in more cohabitation when shelters were limited during the daytime.

## Material and methods

Earwigs were collected under driftwood above the high tide line on beaches on San Juan Island, WA with permission of the University of Washington’s Friday Harbor Laboratories. We only used adults in our trials that were not guarding nests to control for behavior directly related to parental care. After capture, earwigs were marked with unique color/number bee tags (glued to the pronotum) and housed individually in plastic tubs or glass jars (0.5L) with moistened sand. Each earwig was used in a single cohabitation trial within 48 hours, after which it was frozen for subsequent morphological measurements using Image J 1.48v. Using pictures taken from a digital camera (SONY CCR-DC374) attached to a dissecting scope (Nikon SMZ800), we measured the pronotum and abdominal width (using widest point of 6^th^ abdominal segment as an index of body size; [15]). “Size-matched” individuals were defined as having sixth abdominal segment widths within 5% of each other, while “different-sized” individuals differed by more than 10% [21].

The experimental arenas were clear plastic tubs (15 x 21 x 10 cm) filled 0.5 cm deep with moist sand collected from False Bay, San Juan Island, WA. Shelters consisted of a red-tinted plastic square (5x5 cm) covering a small indentation in the sand. This approach allowed for visual monitoring without disturbance. Between each trial, containers and red-tinted shelters were rinsed with warm water and dried for at least 12h and replenished with new sand. Earwigs were maintained on a natural light/dark regime of approximately 14L:10D, and individuals were monitored at nighttime under red light to minimize disturbance.

We ran two sets of experiments: in the first, each arena had two shelters placed 15 cm apart to examine cohabitation in the absence of shelter limitation; in the second, each arena had only one centrally-located shelter where individuals would have to compete for shelter or cohabitate in close proximity. Trials were coded as cohabitating or not cohabitating, where individuals were considered cohabitating if both individuals had their entire head under the shelter, thus indicating their awareness of other occupants. Within each set of experiments, we released two earwigs that were either: (a) different-sized females, (b) size-matched females, (c) different-sized males, (d) size-matched males, or (e) a size-matched male and female. We released earwigs in the late morning, and photographed and recorded their positions at 12h and 24h after release to determine any differences between periods of peak activity (12h, nighttime) and subsequent establishment of stable positions (24h, daytime).

To determine the main drivers of cohabitation tendencies we ran a generalized linear model (GLM) of our binomial data using all parameters (time, shelter, sex pairings, and relative size) as fixed effects (main effects only GLM). All possible interactions were then tested independently (individual interaction testing). Interactions that were significant (p<0.05) were then included in the final model with the fixed effects (full GLM). Post hoc comparisons using Tukey’s honest significant difference tests were used where appropriate. Additionally, we conducted Chi-squared goodness-of-fit tests to analyze individual behavior within trials. All GLMs were performed using R v3.4.0 in RStudio v1.0.143, whereas all Chi-square tests were conducted in JMP Pro 11.

## Results

**Fig 1:**
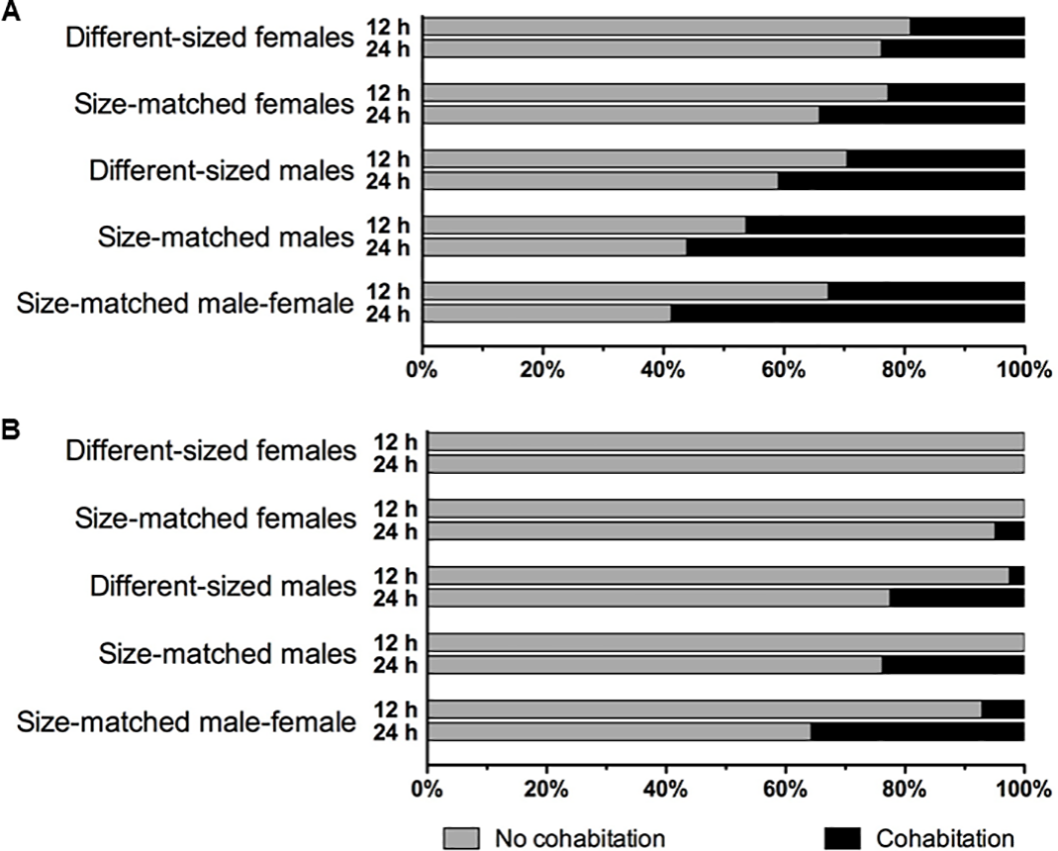
Cohabitation of two earwigs based shelter availability. The bars show the percentages of trials in which two earwigs were found separately (no cohabitation) or under the same shelter (cohabitation) at both 12h and 24h based on the presence of two shelters (A) or one shelter (B).

The cohabitation results of the various pairings are shown in Fig 1. Overall, earwigs were more likely to cohabitate at 24h (daytime) than at 12h (nighttime; Table 1). Regardless of time (12h vs. 24h), earwigs were still more likely to cohabitate with two shelters versus one (Table 1). There was a significant interaction between time and number of shelters present (Table 1), as earwigs with two shelters at 12h are more likely to cohabitate than at 24h with only one shelter (full GLM Tukey HSD: z = -3.269, p = 0.006). Time did not have a significant interaction with sex pairing (individual interaction testing: z = 0.810, p = 0.418).

**Table 1:** Generalized linear models of cohabitation. The categorical fixed effects were time (24h vs. 12h), shelter (1 vs. 2), sex (Males vs. Females vs. Mixed), and size (same-sized vs. different-sized). Model selection was performed using Akaike information criteria (AIC), and only significant interactions were used.

**Full GLM** (AIC = 762.64)				
	Estimate	Standard error	Z value	P value
Time (12h *vs*. 24h)	2.279	0.499	4.567	<0.001
Shelter (1 *vs*. 2)	4.670	0.872	5.357	<0.001
Sex (males *vs*. female *vs*. mixed)	2.588	0.756	3.423	<0.001
Size (size-matched *vs*. different-sized)	0.457	0.212	2.161	0.0306
Time*Shelter	-1.699	0.540	-3.145	0.0017
Shelter*Sex	-1.720	0.793	-2.170	0.0300
**Main effects only** (AIC = 779.16)				
	Estimate	Standard error	Z value	P value
Time (12h *vs*. 24h)	0.936	0.184	5.078	<0.001
Shelter (1 *vs*. 2)	1.791	0.202	8.862	<0.001
Sex (males *vs*. female *vs*. mixed)	1.102	0.217	5.091	<0.001
Size (size-matched *vs*. different-sized)	0.458	0.210	2.179	0.0293

Males and females differed in cohabitation. Females were less likely than males to cohabitate with conspecifics (main effects only Tukey HSD: z = 5.091, p < 0.0001) but were more likely to cohabitate with a male than another female (main effects only Tukey HSD: z = 6.001, p < 0.0001). Interestingly, males were equally likely to cohabitate with another male or a female (main effects only GLM Tukey HSD: z = 2.196, p = 0.071). Sex differences were also apparent in same-sex trials, as pairs of females were less likely to cohabitate than pairs of males (full GLM Tukey HSD: z = 3.423, p < 0.001). There was also a significant interaction between sex pairings and number of shelters (full GLM: z = -2.17, p = 0.030). Specifically, intrasexual pairs of males were less likely to cohabitate with one shelter than mixed-sex pairs with two shelters (full GLM Tukey HSD: z = -6.238, p < 0.0001), and intrasexual pairs of males were more likely to cohabitate with two shelters than mixed-sex pairs with one shelter (full GLM Tukey HSD: z = -3.045, p = 0.028). Furthermore, females with two shelters are more likely to cohabitate than males with one shelter (full GLM Tukey HSD: z = -3.496, p = 0.006), but show identical behavior as mixed-sex pairs with one shelter (full GLM Tukey HSD: z = -0.723, p = 0.979). Additionally, females dominated males in mixed-sex pairs when only one shelter was available, as the female was the lone occupant in 70% of those trials that did not result in cohabitation (χ^2^ = 4.615, df = 1, p = 0.032).

Relative size affects cohabitation, as earwigs are less likely to cohabitate if they are different-sized (full GLM: z = 2.161, p = 0.031). There was no interaction between size and time (individual interaction testing: z = -0.379, p = 0.705), shelter (z = 1.806, p = 0.071), nor sex (z = -0.407, p = 0.684). Overall, among different-sized intrasexual pairs, larger individuals of both sexes were more likely to occupy the shelter at 24h (77% in males: χ^2^ = 9.857, df = 1, p = 0.0017; 84% in females: χ^2^ = 21.404, df = 1, p < 0.0001), and there were no sex differences in this size-based advantage (χ^2^ = 0.462, df = 1, p = 0.497).

## Discussion

Overall, we found that time of day, shelter availability, sex pairings and relative size all affect cohabitation frequency in *A*. *maritima*. Specifically, we found that individuals cohabitated more at 24h than at 12h, that increasing shelter availability increased cohabitation, females were more resistant to cohabitation, and larger individuals were more likely to occupy shelters over smaller individuals.

We initially anticipated finding differences in cohabitation between the 12h and 24h time periods due to changes in familiarity; however, based on the close proximity of individuals observed during daytime field collections, our lack of cohabitation at 12h likely arose from the fact that our 12h time-point was at night, during their peak period of activity (e.g., foraging for food and looking for mates), whereas the 24h check was in the morning during a period of quiescence. Furthermore, since we know that dominance, at least among pairs of males, is usually established within 4 minutes [15], it seems unlikely that dominance among the participants had not been established by the 12h mark.

We expected shelter limitation to increase the frequency of cohabitation, but this was not the case. In fact, cohabitation was rarer with only one shelter—occurring less than 24% of the time overall—in addition to a complete lack of cohabitation among different-sized females. This aversion to cohabitation suggests that limited resources intensified competition thus leading to elevated levels of aggression and less tolerance of rivals. Our results showing that earwigs were more likely to cohabitate at 12h with two shelters (high activity, no habitat limitation) than at 24h with one shelter (low activity, limited habitat) highlights how limited shelters intensify competition. Although shelter seems paramount as *A*. *maritima* is always hidden under driftwood during the day, our study suggests that shelter limitation may increase aggression and lead to less cohabitation than expected. As a result, the distribution and density of suitable shelters in a given location are likely to affect competitive interactions that may, in turn, lead to differences between populations.

Overall, cohabitation did not occur as frequently among pairs of females as between pairs of males, indicating aggression may be higher in females than males. Indeed, we had predicted a greater intolerance of cohabitants by females given that high levels of female aggression among nesting mothers reduce offspring mortality from predation by conspecifics [22]. It follows that selection should favor females who behave aggressively and avoid aggregations while nesting, although we do not know if this aggression is affected by mating or nesting status. Females are usually larger than males, and they often engage in violent battles that result in death [16]. Males, on the other hand, generally partake in less aggressive encounters that involve non-lethal pinches instead of deadly strikes [14,15,23]. These male-male interactions are less likely to involve injuries and may establish a dominance hierarchy that reduces the necessity to waste energy on additional aggression [24,25]. These behavioral differences may explain our findings of differing cohabitation among sexes.

Size-matched male-female pairs initially were unlikely to cohabitate, but were more tolerant of cohabitation later, regardless of the number of shelters. When comparing these size-matched intersexual pairs with intrasexual ones, it is clear that females were the ones changing their behavior to be more tolerant of cohabitating with a size-matched male, perhaps due to interest in mating. These results also indicate that females are more aggressive than males, as females were more likely than males to be the sole occupant of a single shelter in cases where they did not cohabitate. Males were equally likely to cohabitate regardless of whether the other individual was male or female, providing further evidence that females dictate cohabitation rates. As intrasexual pairs with two shelters had a higher or similar likelihood of cohabitating than intersexual pairs with only one shelter, it seems that habitat limitation may play a larger role in tolerance of cohabitants than the sex of cohabitants. This result provides further evidence that habitat limitation reduces tolerance of conspecifics in the maritime earwig.

Size is known to have a strong effect on competitive interactions in *A*. *maritima*, seen with larger males winning contests for food [15]. Our results demonstrate that, when competing for a single shelter, both larger males and larger females dominated their smaller intrasexual counterparts for sole occupancy of a single shelter at 24h, which confirms previous results regarding competition for potential mates [21]. Interestingly, earwig pairs were less likely to cohabitate if there was a mismatch in size, which indicates that size-matched individuals, who are likely to have a similar fighting ability, are more likely to accept cohabitation rather than continue a long battle. The overall reduction in aggressive behavior among size-matched individuals may serve to avoid conflict with same-sex neighbors or to find a mate [17]. This study only observed positions at two important time points, but future continuous observations would greatly increase our understanding of how relative size and sex influences the ratio of aggressive behaviors during encounters. Video recordings of interactions will likely provide insight into the mechanism by which dominance is established, the key traits of the participants that lead to cohabitation, and the typical sequence of behaviors that result in different levels of tolerance [26,27]. It will also be important to examine how these interaction dynamics translate to larger groups, particularly since these earwigs live in close quarters where all individuals possess weaponry in the form of forceps. Previous studies on other earwig species have shown that they have aggregation pheromones that cause gregarious behavior within and between species [28–31]. Although the proximate mechanism of aggregation may involve such a pheromone in *A*. *maritima*, our results show that habitat abundance leads to increased cohabitation while habitat limitation hampers group living. This suggests that other factors, such as mating opportunities or potential food sources via cannibalism [4,16], may be important driving forces behind aggregations in the maritime earwig.

In conclusion, our results show that pairs of maritime earwigs generally prefer not to cohabitate, with females having a stronger aversion to cohabitation than males. Access to shelter plays a big role in cohabitation tendencies in that our two-shelter trials lead to more cohabitation than one-shelter trials, which indicates that factors beyond habitat limitation are responsible for aggregative behavior in *A*. *maritima*. Size, as expected, plays a large role in determining dominance, and mismatched individuals are less likely to cohabitate, probably due to aggression and superior fighting ability by the larger individual. Lastly, the lower levels of cohabitation at 12h appear to reflect their higher activity levels at night, whereas the patterns at 24h are likely to mirror the patterns we have observed during our daytime field observations. In future studies, it would be important to determine the daily modulation of activity levels and site fidelity in the field. While this work provides insight into the factors that may influence the distributional patterns, continuous monitoring of individuals, particularly in larger aggregations, must be done to understand how inter and intrasexual aggression affect group dynamics in the field.
